# Cross-protection induced by Japanese encephalitis vaccines against different genotypes of Dengue viruses in mice

**DOI:** 10.1038/srep19953

**Published:** 2016-01-28

**Authors:** Jieqiong Li, Na Gao, Dongying Fan, Hui Chen, Ziyang Sheng, Shihong Fu, Guodong Liang, Jing An

**Affiliations:** 1 Department of Microbiology, School of Basic Medical Sciences, Capital Medical University, Beijing 100069, PR China; 2 State Key Laboratory of Infectious Disease Prevention and Control, National Institute for Viral Disease Control and Prevention, Chinese Center for Disease Control and Prevention, Beijing 100052, China; 3 Collaborative Innovation Center for Diagnosis and Treatment of Infectious Diseases, Hangzhou, 310003, China; 4 Center of Epilepsy, Beijing Institute for Brain Disorders, Beijing China

## Abstract

Dengue viruses (DENVs) and Japanese encephalitis virus (JEV) are closely related mosquito-borne flaviviruses that cause very high global disease burdens. Although cross-reactivity and cross-protection within flaviviruses have been demonstrated, the effect of JEV vaccination on susceptibility to DENV infection has not been well elucidated. In this study, we found that vaccination with the JEV inactivated vaccine (INV) and live attenuated vaccine (LAV) could induce cross-immune responses and cross-protection against DENV1-4 in mice. Despite the theoretical risk of immune enhancement, no increased mortality was observed in our mouse model. Additionally, low but consistently detectable cross-neutralizing antibodies against DENV2 and DENV3 were also observed in the sera of JEV vaccine-immunized human donors. The results suggested that both JEV-LAV and JEV-INV could elicit strong cross-immunity and protection against DENVs, indicating that inoculation with JEV vaccines may influence the distribution of DENVs in co-circulated areas and that the cross-protection induced by JEV vaccines against DENVs might provide important information in terms of DENV prevention.

Dengue viruses (DENVs), members of the family Flaviviridae, cause one of the most widespread mosquito-borne diseases in tropical and subtropical countries. DENVs, which are transmitted by Aedes aegypti and Aedes albopictus, cause dengue fever (DF) and severe dengue. Four closely related but antigenically distinct serotypes have been identified, namely DENV1-4. An estimated 390 million dengue infections occur each year, and 96 million are clinically apparent, a rate that is three times higher than that reported in 2009^1^,^2^. Recently, two epidemics have emerged in southern Asia and another one in the United States^3^. Moreover, the logarithmic rate at which DF increases in China over the past 4–5 years also highlights the urgency for Chinese to tackle DF endemic^4^. Notably, Guangdong province in China experienced a surge in DF cases in 2014, with the total number of cases exceeding 40,000, which is 60 times the number of infections compared with the number in 2013^5^. Therefore, dengue has evolved from a sporadic disease into a major public health problem, with broader geographical distribution, elevated case numbers and increased disease severity^6^. However, currently, there is still no available vaccine that provides balanced protection against DENV1-4, although several vaccines are being developed^7^,^8^,^9^.

Japanese encephalitis virus (JEV), which also belongs to the Flaviviridae family, is genetically and antigenically closely related to DENVs. JEV and DENVs share 54.3% amino acid sequence homology in the envelope [E] protein^10^. JEV co-circulates with DENVs in the Indian subcontinent and in Southeast Asia. In contrast with DENVs, there are several vaccines for JEV, including a live attenuated vaccine (LAV) and inactivated vaccines (INVs). The vaccine strain SA14-14-2 is currently the only JEV-LAV available, and it has been used with great success for decades in mainland China and more recently in other Asian countries^11^. One INV is the formalin-inactivated JEV vaccine, which is purified from infected mouse brain (BIKEN or JE-VAX) and is based on either the Nakayama or Beijing-1(P1) strains; it is currently the only WHO-recommended vaccine used worldwide. Moreover, the recently developed Vero cell-derived inactivated vaccine containing the purified, inactivated JEV strain SA14-14-2 has been approved (IXIARO); it is mainly used in Australia and in European countries^12^. In a previous study, we characterized the immune response and protective efficacy induced by the INV, LAV and the DNA vaccine candidate pCAG-JME (expressing JEV prM-E protein) in mice, and we reported that the LAV conferred 100% protection against JEV infection and resulted in the generation of high levels of specific anti-JEV antibodies and cytokines^13^. Therefore, we hypothesized that JEV vaccines that are licensed may confer protection against closely related flaviviruses that have no available vaccines, such as DENVs.

JEV and DENV exhibit significant serological cross-reactivity, which can complicate the assessment of the relative burdens of each virus in co-epidemic areas and their possible interactions^14^,^15^. Furthermore, understanding the potential interactions between DENV and JEV is important in terms of public health research because JEV continues to co-circulate with DENV in Southeast Asia, the area with the highest burden of DENV illness and high JEV vaccination coverage. For many years, it has been known that vaccine inoculation will provide cross-protective immunity against heterologous viruses belonging to the same group. Generally, flaviviruses can be classified into various subgroups based on their transmission vectors. Investigations of cross-protection have mainly focused on the same subgroups^16^,^17^,^18^, such as cross-protection between JEV and West Nile virus (WNV). Tesh *et al.* have reported that immunization with the live attenuated SA14-2-8 strain of JEV protected against WNV^19^. Tarr GC *et al.* have reported that immunization with DENVs could protect against JEV, St. Louis encephalitis and WNV^20^,^21^,^22^. However, a few studies have investigated cross-protection between the different subgroups. In particular, only limited evidence is available regarding the effect of JEV vaccination on DENV infection. Thus, given the ambiguous findings from previous studies, understanding the influence of preexisting JEV immunity on DENV infection is important for the development of preventive strategies against dengue, such as the evaluation of new DENV vaccines.

Thus, this study aimed to characterize the cross-immunity elicited by JEV-INV and JEV-LAV against DENV infection in mice and JEV-vaccinated human subjects. We observed some cross-immunity and cross-protection between JEV and DENVs. Our results will provide insight into the development of a novel preventive strategy against DENVs, as well as an analysis of the potential influence of JEV vaccination on the distribution of DENV infection.

## Results

### The levels of cross-reactive antibodies to DENV1-4 after immunization with JEV vaccines

**Figure 1:**
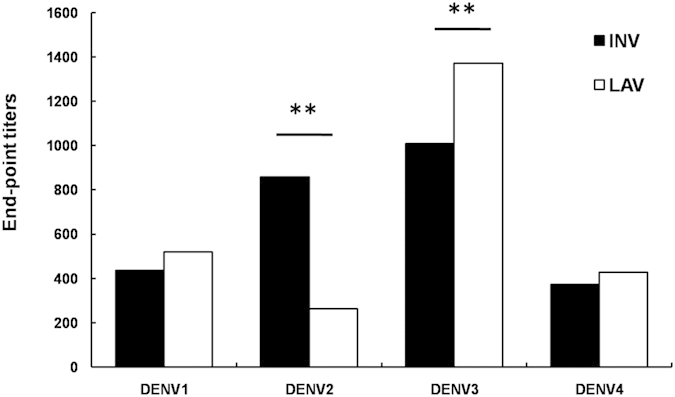
The end-point titers of the cross-reactive antibodies were assayed with ELISA and determined as the reciprocal of the highest dilution providing an optical density (OD) twice that of the PBS-immune serum (n = 10). Sera were collected from immunized mice three weeks after the final immunization. The results shown above are the geometrical mean titers.

The levels of cross-reactive antibodies to DENV1-4 induced by the JEV vaccines are shown in Fig. 1. The end-point titers of IgG cross-recognizing the DENV1 antigen were 1:519 and 1:436 in the JEV-LAV and JEV-INV groups, respectively, with no significant between-group differences. However, the response pattern of the cross-reactive IgG titers to DENV2 differed from that to DENV1: the titer (1:857) observed in the INV group was significantly higher than that observed (1:264) in the JEV-LAV group (p < 0.01). The JEV-LAV-immunized mice developed cross-IgG to DENV3, with titers at 1:1372, which were significantly higher than the 1:1008 level detected in the JEV-INV group (P < 0.05). The cross-IgG responses to DENV4 in the JEV-LAV and JEV-INV groups were 1:424 and 1:373, respectively, with no between-group differences (p > 0.05). This indicated that both JEV-LAV and JEV-INV induced cross-IgG against DENVs.

### The levels of cross-neutralizing antibodies to DENV1-4 after immunization with JEV vaccines

To detect the levels of cross-neutralizing antibodies (NAbs) in the sera of immunized mice, samples collected after the final immunization. As shown in Table 1, the cross-NAb titer in the phosphate buffered saline (PBS) group did not exceed 1:10. Notably, the cross-NAb titers to DENV1 in the JEV-LAV group were 1:113, which was significantly higher compared to the JEV-INV group (1:30, p < 0.01). Similarly, JEV-LAV elicited higher cross-NAb levels to DENV3 and DENV4, with titers of 1:59 and 1:71, respectively, compared with the JEV-INV group, in which only 1:23 and 1:17 cross-NAb titers were detected. There were significant between-group differences in the cross-NAb titers against DENV3 or DENV4 (p < 0.05). However, consistent with the cross-reactive IgG response to DENV2, the NAb titers to DENV2 in the JEV-LAV-immunized mice were 1:74, which was also significantly lower than that (1:403, p < 0.05) in the JEV-INV-immunized mice.

### Cell-mediated immune response to DENV 1-4 after immunization with JEV vaccines

**Figure 2:**
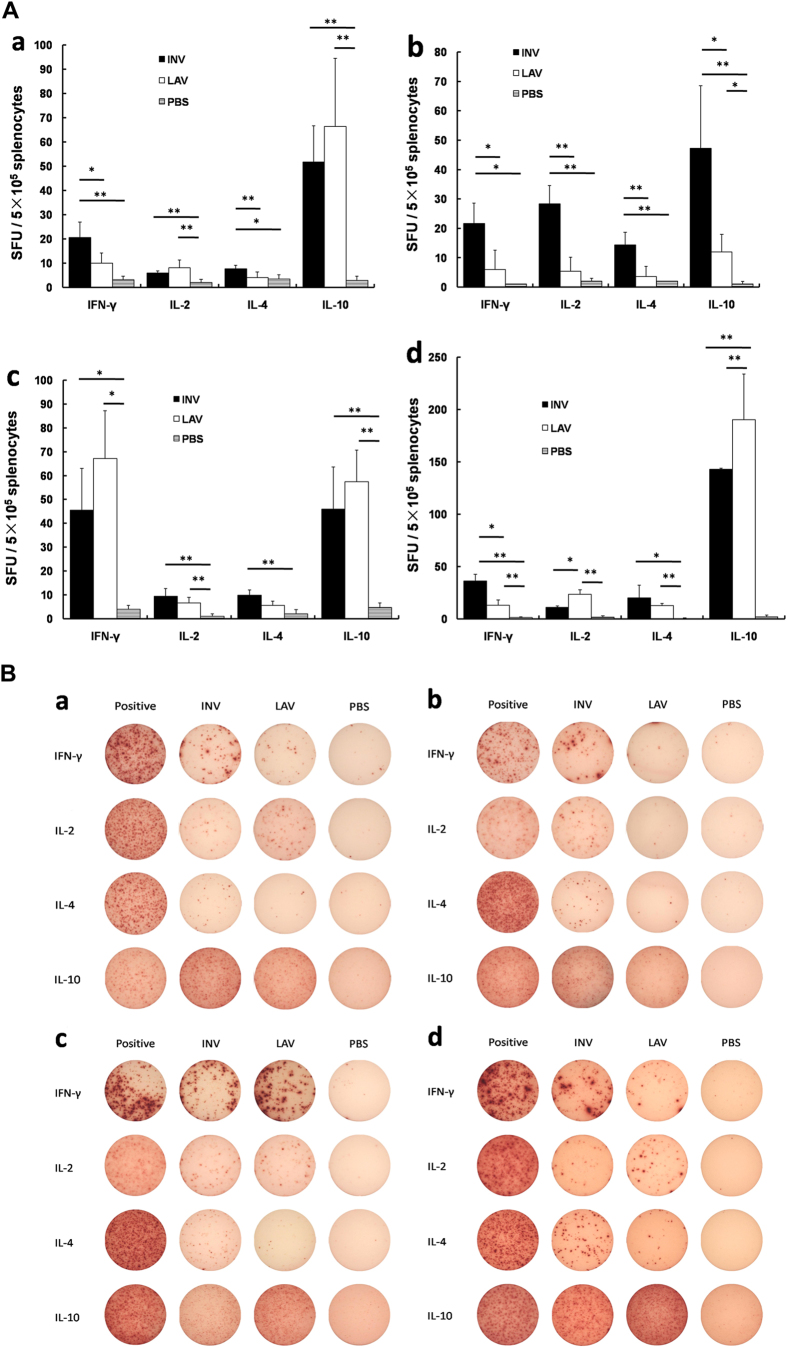
Splenocyte-secreted cytokines upon DENV1-4 antigen stimulation were detected by ELISPOT (n = 5). (**A**) The numbers of cytokine-positive cells are expressed as spot-forming units (SFU)/5 × 10^5^ cells after background subtraction. (a) DENV1 antigen stimulation (*p < 0.05, **p < 0.01). (b) DENV2 antigen stimulation (*p <  0.05, **p < 0.01). (c) DENV3 antigen stimulation (*p < 0.05, **p < 0.01). (d) DENV4 antigen stimulation (*p < 0.05, **p < 0.01). (**B**) a representative raw ELISPOT data in the format of scanned image, a–d: stimulated by DENV1-4 antigens, respectively.

After the final vaccination, the splenocyte-derived cytokine levels in each group were determined, and the results are shown in Fig. 2. When stimulated with DENV1 antigen, significantly elevated levels of the four cytokines (i.e., interferon-γ (IFN-γ), interleukin (IL)-2, IL-4, and IL-10) were observed in the JEV-INV and JEV-LAV vaccination groups compared with the control group (Fig. 2A-a,2B-a, p < 0.05 or 0.01). However, when stimulated with DENV2, there were no obvious changes in the JEV-LAV group; the levels of the four cytokines were all significantly increased in the JEV-INV group compared with the control groups (Fig. 2A-b,2B-b, p < 0.05 or 0.01). Similar to DENV1, stimulation with DENV3 (Fig. 2A-c,2B-c) or DENV4 (Fig. 2A-d,2B-d) profoundly increased the cytokine levels of IFN-γ, IL-2, IL-4 and IL-10 in the JEV-INV and JEV-LAV groups compared with the control group (p < 0.05 or 0.01).

Generally, IFN-γ and IL-2 are the biomarkers for Th1-type immune responses, and IL-4 and IL-10 are the biomarkers for Th2-type immune responses. The study results indicate that the Th1- and Th2-type immune responses were induced following the vaccination (with both vaccines) when stimulated with DENV1, DENV3 and DENV4. However, when stimulated with DENV2, JEV-INV induced the Th1- and Th2-type immune responses, while JEV-LAV did not induce any evident cross-humoral or cellular immune responses.

### Mouse challenge experiments

**Figure 3:**
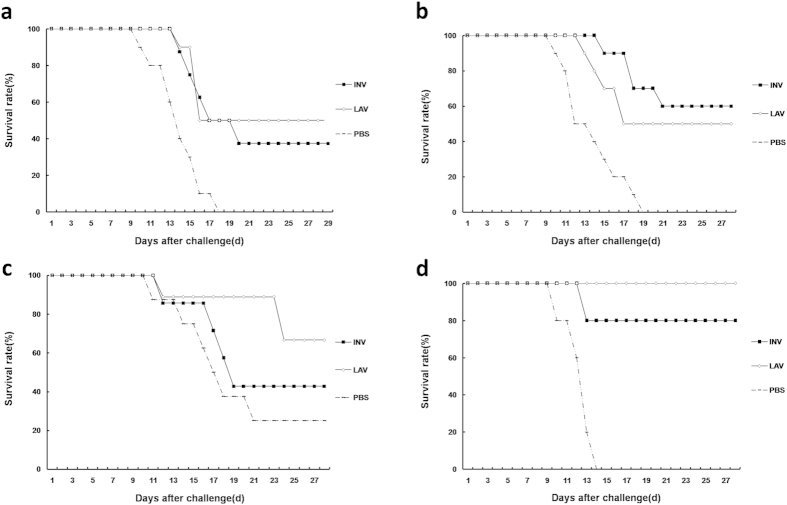
The immunized mice were intracerebrally (i.c.) challenged with a lethal dose of DENV 1-4. The results are expressed as the survival rates. (**a**–**d**) Challenged by DENV1-4, respectively.

The survival rates of mice in each group following challenge with a lethal dose of DENVs are shown in Fig. 3 and Table 1. Consistent with the enzyme-linked immunosorbent assay (ELISA), plaque reduction neutralization test (PRNT)50 and ELISPOT results, the JEV-LAV- and JEV-INV-immunized mice were partially protected from the DENV1 challenge, with 50% and 40% survival rates, respectively. The JEV-LAV and JEV-INV immunization also induced cross-protection against DENV2, and the survival rates in the two groups were 50% and 60%, respectively. Similarly, the mice in the JEV-LAV and JEV-INV groups were cross-protected from DENV3 challenge, with 67% and 43% survival rates, respectively. Surprisingly, the mice in JEV-LAV group were completely protected from the DENV4 challenge, with a 100% survival rate, while the survival rate of the mice in the JEV-INV group was 80%. All mice in the PBS-immunized group had a 100% mortality rate when challenged with DENV1, 2 and 4, while a 75% mortality rate was observed when they were challenged with DENV3. Importantly, no increased mortality was observed in our mouse model when challenge. These results indicated that both the JEV-LAV and JEV-INV vaccines were able to induce cross-protective immunity against lethal challenge with DENVs.

### The cross-NAb levels to DENV2 and DENV3 in the sera of subjects vaccinated with JEV vaccines in a non-DENV endemic region

Sera samples from human subjects vaccinated with JEV-INV or JEV-LAV, which contain high NAb levels to JEV (Tables S1 and S2), were collected to examine cross-NAb levels to DENV2 and DENV3. As shown in Table 2, the cross-NAb titers (PRNT50) to DENV2 in the sera samples from the JEV-INV and JEV-LAV groups ranged from 1:20 to 1:80, with average titers of 1:40 for both groups, indicating no between-group difference in the cross-NAb titers from the JEV-INV and JEV-LAV vaccinated subjects. Interestingly, the cross-NAb titers to DENV3 in both groups showed several differences: in the JEV-INV group, the titers with 1:20 to 1:80 were observed in most of the samples (90%), and only the remaining 10% of samples (3 cases) showed high titers (greater than 1:160); but in the JEV-LAV group, the samples containing 1:20–1:80 NAb titers accounted for 70%, and the remaining 30% of samples showed high titers (1:160 - 1:320, Table 2). The average cross-NAb titers to DENV3 were 1:54 and 1:97 in the JEV-INV and JEV-LAV groups, respectively (Table 2), and there were significant differences between the two groups (p < 0.05). The results indicated that the JEV-LAV vaccination induced higher cross-NAb titers to DENV3 (but not to DENV2) compared with the JEV-INV vaccination.

## Discussion

Viruses in the Flaviviridae family are enveloped and contain a positive-sense, single-stranded RNA genome. All members of the genus Flavivirus are antigenically related, and distinct serocomplexes are defined based on cross-neutralization tests^1^,^2^. Recently, the cross-reactivity in immune responses to flavivirus infection raised an interesting question in terms of vaccine development: does cross-immunity provide cross-protection within the same family? Previous studies have demonstrated cross-protection between members in the same subgroups of the Flaviviridae family^16^,^17^,^18^,^19^,^20^,^21^,^22^. However, there have been limited investigations of cross-protection between different subgroups. JEV and DENVs are members of the Flaviviridae family, but they belong to different serocomplexes. In the present study, we demonstrated a protective effect from the cross-immunity against DENVs, which was induced by the JEV vaccines in the mice. Our results provide important information in understanding the interaction between the Flaviviridae family members which further assist the development of a novel bivalent vaccine against JEV and DENVs, such as recombinant subunit and/or DNA vaccines.

In the current study, JEV-LAV and JEV-INV induced high levels of DENV-cross reactive IgG and NAb that are significant effectors against viral infection. However, the pattern of cross-immunity induced by the JEV vaccines differed among DENV1-4. In DENV1, 3 and 4 cases, JEV-LAV induced a higher trend in the titers of IgG and NAb compared with JEV-INV, whereas the opposite result was observed in DENV2. Consistently, the elevated production of IFN-γ and IL-2 as well as IL-4 and IL-10, markers of the Th1- and Th2-immune responses, respectively, were also shown in the same pattern to the cross-humoral response mentioned above. The results indicated that the characterization of cross-immunity to DENVs induced by JEV-LAV and JEV-INV may be dependent upon DENV serotypes. The cross-immunity induced by JEV-LAV was dominant in the DENV1, 3 and 4 cases, whereas the JEV-INV-induced cross-immunity was dominant for DENV2. However, the mechanisms underlying this phenomenon remain unclear and clarifying this issue in further study will be helpful for developing vaccine to DENV.

Furthermore, we found that the JEV-LAV and JEV-INV immunized mice were all partially resistant to the DENV1-4 challenge in a protection test, and there were no significant between-group differences in the survival rates for each DENV serotype for the two JEV vaccine groups, although the patterns of cross-immunity to DENVs differed. This result suggested that the cross-immunity induced by JEV-INV and JEV-LAV, including the humoral and cellular immune responses, could protect mice from DENV challenge. Surprisingly, 80% and 100% survival rates were observed in the DENV4 challenge following JEV-INV and JEV-LAV immunization, respectively, indicating effective cross-protection. Previous studies have shown that immunization with infectious JEV or live attenuated JEV induced protective immunity against West Nile encephalitis in hamsters and monkeys^19^,^23^. Our results demonstrated that JEV vaccines also induced cross-protective immunity to DENVs in mice and the different cross-immunity patterns induced by the JEV vaccines did not influence the cross-protection. It is noteworthy that no increased mortality was observed in our mouse model. Moreover, it was reported that antibody to DENV prM is a major component of the enhancement activity and there are only 35% homology in amino acid sequences of prM between DENV and JEV^24^. Therefore, we speculated that anti-prM antibodies elicited by JEV-vaccines may cause less ADE than the anti-prM cross-reactive antibodies induced by DENV infection. Together, this study provides new insight into the development of preventive strategies against DF. However, there are some limitations. One, animal data may not completely reflect the events happened in human. Two, we performed PRNT assay using C6/36 mosquito cells propagated DENV, which may be different from results obtained using Vero cells propagated DENV. It is known that DENV grown *in vitro* in various cell types will give rise to progenitor viruses of slightly different property^24^, thus, the interpretation of PRNT assay results should take viruses used into consideration.

Next, we detected cross-immunity between JEV and DENVs in human subjects vaccinated with JEV-INV or JEV-LAV. The high NAb levels against JEV indicated successful vaccination (Table 2). We found that the recipients developed high levels of cross-NAbs with titers 1:20–1:320 against DENV2 and DENV3, thus further supporting the cross-immunity between JEV and DENVs. The cross-immune response pattern to DENV2 induced by JEV-INV or JEV-LAV vaccination differs from that to DENV3 (Table 2); however, combined with the results of the challenge experiment, there were no significant differences between the JEV-LAV and JEV-INV immunized mice in the cross-protection against DENV2 or DENV3, indicating that effective cross-protection was induced by both JEV vaccines. However, we note that relatively high survival rates and high IgG and NAb titer levels were observed in the JEV-LAV group but not in JEV-INV group when challenged with DENV1, 3 and 4. It has been reported that fewer cases of severe DF were observed during the outbreaks in mainland China in recent years compared with those observed in southeast Asian countries, such as Thailand and Singapore. These results implied that the JEV-LAV vaccination, which has been used in mainland China for more than 20 years, may exhibit a protective effect against DENV1, 3 and 4 infection and/or reduce the disease severity.

The mechanisms underlying the cross-protection between the JEV vaccines and DENVs are not clear, but they are likely associated with common antigens within flaviviruses. The flavivirus genome encodes a single polyprotein precursor encoding three structural proteins (i.e., the capsid [C], pre-membrane [prM], and envelope [E] proteins) and seven nonstructural proteins^25^. Previous studies have demonstrated that domain II of the E protein (EDII), a distinct E protein domain, is able to induce cross-reactive immune responses to members of the flavivirus group^26^,^27^,^28^,^29^,^30^. Moreover, we found that the JEV and DENVs share 54.3% amino acid sequence homology in the E protein and their aa100-114 of E protein (locating at domain II) are identical. Plausibly, the highly conserved epitopes at the fusion loop of EDII might be responsible for the cross-reactivity induced by the JEV vaccine against DENVs. Together, our results strongly indicate that JEV vaccines could induce cross-protection against DENVs. Further identification of the cross-immunization epitopes is important to develop a vaccine against DENVs and/or a novel bivalent vaccine against both DENV and JEV. Currently, cross-NAbs to DENV1-4 from monoclonal antibodies against JEV were successfully screened, and analysis of common epitopes related to cross-immunity is ongoing in our lab.

It is generally known that DENVs threatens the health of more than 2.5 billion people in urban, peri-urban, and rural areas in the tropics and subtropics, particularly in Asia^3^. The changing patterns in the epidemiology of DENV infection may be due to multi-factorial causes^31^,^32^,^33^,^34^,^35^, including human genetics, increased human movement (e.g., movement from rural to urban areas), global warming, vaccination and co-circulation of some viruses^36^,^37^,^38^. Among these causes, vaccination with the related vaccines appears to be impacting the epidemiology of DF in different manners in epidemic areas worldwide. Currently, JEV-LAV and JE-VAX have been used for decades in Southeast Asia, which has the highest burden of DENV illness and high JEV vaccination coverage. In the present study, we demonstrated that JEV-INV and JEV-LAV induced cross-immunity and cross-protection against DENVs in mice. Furthermore, cross-NAbs titers were also detected in the sera of human recipients of JEV vaccination. Importantly no evidence of deleterious immune enhancement was found in both mouse challenge and immune studies. We suggested that JEV-INV and JEV-LAV could induce cross-immunity and provide partial cross-protection to DENVs, indicating that the inoculation of JEV vaccines might influence the distribution of DF in areas where both viruses are co-circulating. In addition, the cross-immunity may be an important factor that should be considered in the evaluation of new DENV vaccines.

In summary, this study demonstrated cross-immunity and cross-protection between JEV and DENVs. Thus, it is logical and worthwhile to identify common epitopes for future development of a novel bivalent recombinant subunit vaccine against DENV and JEV. Meanwhile based on our results, the potential benefit or influence of JEV vaccines on DF epidemiology should be considered in view of their shared geographical areas of co-circulation.

## Material and Methods

### Cells, virus, vaccine, sera and mice

Vero cells were grown in a minimal essential medium (MEM) supplemented with 5% fetal bovine serum (FBS). *Aedes albopictus* C6/36 cells were grown in RPMI1640 supplemented with 10% FBS at 28 °C. The DENV1 (Hawaii), DENV2 (Tr1751), DENV3 (H87) and DENV4 (H241) strains used in all assays were propagated in C6/36 cells. The virus titers and PRNT were determined through plaque assays on the Vero cells.

The live-attenuated SA14-14-2 vaccine was produced by the Chengdu Institute of Biological Products (CDIBP, Chengdu,China), and the inactivated vaccine derived from Vero cells (based on the P3 strain) was produced in China by Liaoning Chengda Biotechnology, Co., Ltd (CDBIO).

Fifty-two serum samples from human recipients vaccinated with JEV-INV or JEV-LAV in a non-DENV epidemic area (Ningxia, China) were provided by the China CDC. There were 30 JEV-INV and 22 JEV-LAV recipients, respectively. All recipients were approximately 2 years old, and the ratio of boys to girls was approximately 1:1. Institutional board approval was obtained from China CDC and written informed consent was obtained from the guardians of the vaccine recipients. The JEV-INV and JEV-LAV vaccination was performed according to the JEV vaccination guidelines: for LAV 1 dose at 8 months old and 1 dose at 2 years old; and for INV 2 doses at 8 months and 1 dose at 2 years old. At 10–14 days after the final vaccination, serum samples were collected to determine the levels of NAbs against JEV and the levels of cross-NAbs to DENVs.

Six-week-old female BALB/c mice, which were purchased from the Academy of Military Medical Sciences (Beijing, China), were maintained in specific-pathogen-free environments. All animal experimentation were approved by and conducted in accordance with the guidelines set by the Institutional Animal Care and the animal ethics committees of Capital Medical University.

### Mouse experiments

For the immunity test, thirty female BALB/c mice (six-week-old) were divided into three groups for immunization with JEV-INV (P3 strain), JEV-LAV (SA 14-14-2 strain) and PBS. For vaccination, the mice were administered a dose corresponding to approximately one-tenth of the dose recommended for human use (three times at two-week intervals)^13^. The mice immunized with PBS served as negative controls. Three weeks after the final immunization, the mice were euthanized for the evaluation of the cross-reactive antibody titers to DENVs, cross-NAb titers in the sera, and cytokines derived from splenocytes stimulated by to DENVs in the different groups. For the protection test, the mice were immunized (as described above), and three weeks after the final immunization, the mice were intracerebrally (i.c.) challenged with a lethal dose of DENV1 or 2 or 3 or 4. The mortality rates of the mice were observed for 28 days following the infection.

### Antibody assay

The mouse serum samples were collected three weeks after the final immunization and then analyzed for the presence of cross-reactive antibodies to DENVs by ELISA, as previously described^39^,^40^. Briefly, the concentrated viral particles of DENV1-4 were diluted and 96-well plates were coated with 10 μg of the concentrated DENV1, 2, 3 or 4 in each well in 100 μl of carbonate-bicarbonate buffer (NaHCO_3_, 18.2 mMNa_2_CO_3_, pH 9.6) at 4 °C overnight. Following washing and blocking, 100 μl of two-fold serial dilutions of the serum samples (from 1:100) was added to each well and incubated at 4 °C overnight. Then, 100 μl of diluted horseradish peroxidase (HRP)-conjugated goat anti-mouse IgG (1:3000, KPL) was added to each well, and the color was generated by adding the substrate solution of o-phenylenediamine (OPD). Absorbance (at 492 nm) was measured on a microplate reader (Multiskan, Mk3, Thermo). The end-point titers of anti-DENV antibodies were determined as the reciprocal of the highest dilution, providing an optical density twice that of the PBS-immune serum.

### Plaque reduction neutralization test (PRNT)

To determine the NAb titers in the sera of the immunized BALB/c mice and the human recipients vaccinated with JEV vaccines, heat-inactivated sera samples were serially diluted two-folds from 1:10 to 1:5120 with MEM containing 2% FBS. Then, 500 μl of the serial dilutions were incubated with an equal volume of virus solution (DENV1 or 2 or 3 or 4) containing approximately 500 PFU at 37 °C for 1 h. Then, 200 μl of the mixture was added to duplicate wells of the Vero cell monolayer in a 24-well plate, and each well contained approximately 100 PFU of the virus. The plate was incubated at 37 °C for 1 h. After washing two times, the infected Vero cells were overlaid with MEM containing 5% FBS and 1.2% methyl-cellulose. The plates were incubated at 37 °C in 5% CO_2_ for 7–9 days, when the plaque formation could be confirmed. The NAb titers were recorded as PRNT 50 i.e., the reciprocal of the maximum dilution of serum that yielded a 50% plaque reduction compared with that of the virus incubated with the sera of the control mice. Then geometric mean titers (GMT) of PRNT50 were calculated using a standard equation.

### Cytokine ELISPOT assays

To examine the cross-cellular immune response, splenocytes from the immunized mice were obtained after the final immunization. The splenocyte-derived cytokines (i.e., IFN-γ, IL-2, IL-4, and IL-10) were enumerated with an ELISPOT assay, according to the manufacturer’s instructions^39^,^40^. Briefly, 96-well filtration plates (Millipore, USA) were coated with 50 μl (10 μg/ml) of anti-mouse IFN-γ, IL-2, IL-4 or IL-10 capture antibodies at 4 °C overnight. The next day, the plates were blocked with sterile RPMI1640 containing 10% FBS and 1% BSA at 37 °C for 2 h. Then, a total of 5 × 10^5^ splenocytes were added to each plate well and stimulated with 50 μg concentrated viral particles of DENV1-4 s at 37 °C for 48 h, respectively. After stimulation, the cells were incubated with biotin-conjugated Abs and streptavidin-HRP. The cells were also incubated with ConA (5 μg/ml, Sigma) as a positive control and with RPMI1640 medium alone as a negative control. The spots numbers in negative and positive control were traditionally >20 and <3 respectively. The number of each cytokine-secreting cell was determined by the automated ELISPOT reader and analyzed with ImmunoSpot image analyzer software v4.0.

### Statistical analysis

Statistical analysis of ELISA, PRNT and ELISPOT were performed using one-way ANOVA with SPSS 16.0 software. P values below 0.05 were considered statistically significant. The survival rate of mice was analyzed by log-rank test.

## Additional Information

**How to cite this article**: Li, J. *et al.* Cross-protection induced by Japanese encephalitis vaccines against different genotypes of Dengue viruses in mice. *Sci. Rep.* **6**, 19953; doi: 10.1038/srep19953 (2016).

## Supplementary Material

Supplementary Information

## Figures and Tables

**Table 1:** The levels of cross-NAb and survival rates in JEV vaccines-immunized BALB/c mice.

Types	Groups	Neutralizing antibody titers^a^	P		Survival %^b^ (No. of survivors/No. of challenged)	P	
DENV1	INV	1:30	0.006^c^	0.018^d^	40(4/10)	0.612^c^	0.007^d^
	LAV	1:113	—	0.002^e^	50(5/10)	—	0.002^e^
	PBS	<1:10	—	—	0(0/10)	—	—
DENV2	INV	1:403	0.003^c^	0.002^d^	60(6/10)	0.434^c^	0.000^d^
	LAV	1:74	—	0.000^e^	50(5/10)	—	0.006^e^
	PBS	<1:10	—	—	0(0/10)	—	—
DENV3	INV	1:23	0.000^c^	0.000^d^	43(3/7)	0.245^c^	0.435^d^
	LAV	1:59	—	0.000^e^	67(6/9)	—	0.043^e^
	PBS	<1:10	—	—	25(2/8)	—	–
DENV4	INV	1:17	0.028^c^	0.006^d^	80(8/10)	0.146^c^	0.000^d^
	LAV	1:71	—	0.018^e^	100(10/10)	—	0.000^e^
	PBS	<1:10	—	—	0(0/10)	—	—

^a^The geometrical mean neutralizing antibody titers in sera of pre-challenged mice.

^b^Mice were challenged i.c. with DENVs 3 weeks after the final immunization, and then monitored daily for 28 days. The survival rates were determined as 100 × (number of survivors)/(numbers of challenged).

^c^P value was calculated between INV and LAV.

^d^P value was calculated between INV and PBS.

^e^P value was calculated between LAV and PBS.

**Table 2:** The cross-NAb titers to JEV, DENV2 or DENV3 in human subjects vaccinated by JEV vaccines.

Titer	JEV(PRNT90)		DENV2(PRNT50)		DENV3(PRNT50)	
	INV(N = 30)	LAV(N = 22)	INV(N = 30)	LAV(N = 22)	INV(N = 30)	LAV(N = 22)
<1:10	3(10%)	2(9.1%)	—	—	—	—
1:10	2(6.7%)	2(9.1%)	—	—	—	—
1:20	4(13.3%)	3(13.6%)	7(23.3%)	4(18.2%)	1(3.3%)	2(9.1%)
1:40	4(13.3%)	3(13.6%)	15(50%)	8(36.4%)	21(70%)	2(9.1%)
1:80	8(26.7%)	2(9.1%)	6(20%)	9(40.9%)	5(16.7%)	10(45.5%)
1:160	4(13.3%)	3(13.6%)	1(3.3%)	1(4.5%)	0	4(18.2%)
1:320	—	—	1(3.3%)	0	3(10%)	4(18.2%)
>1:320	5(16.7%)	7(31.8%)	—	—	—	—
GMT^a^	—	—	1:40	1:40	1:54	1:97
P^b^	—		P > 0.05		P < 0.05	

^a^GMT geometric mean titer.

^b^*P* values were obtained by ANOVA when comparing the cross-NAb titers between INV and LAV groups. *P* values of <0.05 were considered to be significant.
