# Supplementary material for: Cross-protection induced by Japanese encephalitis vaccines against different genotypes of Dengue viruses in mice
Source: Sci Rep. 2016 Jan 28;6:19953. doi: 10.1038/srep19953 (PMC4730143; doi:10.1038/srep19953)
Supplement: Supplementary Information [file srep19953-s1.pdf]

Cross-protection induced by Japanese encephalitis vaccines against different genotypes of Dengue viruses in mice

Jieqiong Li[1], Na Gao[1], Dongying Fan[1], Hui Chen[1], Ziyang Sheng[1], Shihong Fu[2,3],Guodong Liang[2,3], Jing An[1,2,4]

Supplementary Table1 The NAb titers to JEV, DENV2 or DENV3 in human subjects of each person vaccinated by JEV-INV

No. INV	Neutralizing antibody titers		
	JEV(PRNT90)	DENV2(PRNT50)	DENV3(PRNT50)
1	>1:320	40	80
2	>1:320	20	40
3	>1:320	40	40
4	>1:320	40	40
5	>1:320	20	80
6	1:160	160	40
7	1:160	40	40
8	1:160	20	40
9	1:160	40	80
10	1:80	20	40
11	1:80	40	40
12	1:80	80	40
13	1:80	20	40
14	1:80	80	80
15	1:80	40	40
16	1:80	40	40
17	1:80	20	40
18	1:40	80	40
19	1:40	40	80
20	1:40	40	40
21	1:40	40	40
22	1:20	40	40
23	1:20	40	20
24	1:20	40	320
25	1:20	80	40

26	1:10	80	40
27	1:10	40	320
28	<1:10	20	40
29	<1:10	80	40
30	<1:10	320	320
GMT [a]	—	40	54.014

a: GMT geometric mean titer.

Supplementary Table 2 The NAb titers to JEV, DENV2 or DENV3 in human subjects of each person vaccinated by JEV-LAV

No. LAV	Neutralizing antibody titers		
	JEV(PRNT90)	DENV2(PRNT50)	DENV3(PRNT50)
1	>1:320	80	160
2	>1:320	160	80
3	>1:320	80	20
4	>1:320	40	320
5	>1:320	20	20
6	>1:320	40	80
7	>1:320	40	80
8	1:160	80	80
9	1:160	80	80
10	1:160	20	40
11	1:80	40	40
12	1:80	40	160
13	1:40	40	320
14	1:40	40	160
15	1:40	80	320
16	1:20	80	80
17	1:20	80	160
18	1:20	40	320
19	1:10	80	80
20	1:10	20	80
21	<1:10	80	80
22	<1:10	20	80
GMT [a]	—	40	96.64716

a: GMT geometric mean titer.
